# Patient Perception of Acute Pain Management: Data from Three Tertiary Care Hospitals

**DOI:** 10.1155/2017/7459360

**Published:** 2017-03-28

**Authors:** Elsy Ramia, Soumana C. Nasser, Pascale Salameh, Aline Hanna Saad

**Affiliations:** Department of Pharmacy Practice, School of Pharmacy, Lebanese American University, P.O. Box 36 (S23), Byblos, Lebanon

## Abstract

*Introduction*. The primary objectives of this study were to assess patients' description of their acute pain intensity; patients' attitude towards their pain management during hospitalization; and their overall satisfaction with pain treatment.* Methodology*. A cross-sectional questionnaire-based study was conducted between October 2014 and March 2015 in three medical centers in Lebanon. All participants' responses were reported using descriptive statistics. The association between categorical variables was evaluated using Pearson *χ*^2^ test or Fisher's exact test where the expected cell count was < 5.* Results*. A total of 119 women on the maternity services and 177 patients on the orthopedic services were surveyed. Around 50% of obstetric and 37% of orthopedic patients reported pain to be severe at its highest intensity. In maternity and orthopedic patients, respectively, unfavorable practices included pain not being assessed prior to pain medication administration (19.3% and 30.5%), having to wait for ≥30 minutes before getting the pain medication (14.2% and 11.3%), and pain score not being documented on medical chart (95% and 93.2%). Surprisingly, 94.1% of the maternity and 89.2% of orthopedic patients were satisfied to strongly satisfied with their pain management.* Conclusion*. Pre- and postoperative pain remain a prevalent problem that requires a consensus and joint efforts for improvement.

## 1. Introduction

Pain-related position statements and published clinical guidelines consider pain as the “fifth vital sign” that requires a holistic management approach [1–5]. Pain management encompasses a comprehensive screening and assessment; collaborative care planning comprising patient and family input; efficacious treatment resulting in adequate pain relief, low functional interference, and few adverse effects; and frequent reassessments of patient responses to treatment [5]. Patient's right to involvement in all aspects of his/her pain management is promoted by governing organizations on pain, clinical guidelines, and healthcare institutions [1–6]. Based on the recommendations of the American Academy of Pain Medicine, the American Pain Society, and the American Society of Anesthesiologists, healthcare institutions have the responsibility to ensure the patients' right to optimal pain management [1–3]. Moreover, the Joint Commission (JC) commits healthcare organizations to pain management as an integral component of care as detailed in their standards on Patients' Rights and Ethics [1–6].

Pain management is a major concern for patients. Patients' perception of pain care became a vital criterion and a relevant outcome measure for healthcare institutions [7]. Furthermore, patients' satisfaction with treatment is crucial to measure performance and success of healthcare institutions [8]. Patients expect to receive optimal pain management resulting with prompt and effective pain control and few adverse effects from pain or its treatment [7, 9]. In addition to adequate pain relief, patient's overall satisfaction depends on multiple factors including delivering a quick intervention, engaging patients in their own care, encouraging their communication of pain, interacting with the healthcare provider, and establishing a trust-based relationship [10–12].

Suboptimal pain control has been frequently reported in acute care settings and documented to negatively impact patients' health, delay recovery, increase postoperative morbidity, and reduce patient satisfaction [2, 4, 13, 14]. It also increases the risk of developing chronic pain resulting in higher use of healthcare resources and costs [15, 16]. According to the National Center for Health Statistics, 46 million Americans experience acute surgical pain following surgeries in the in-patient settings [17]. In fact, results from a national survey conducted among adult patients who had undergone surgical procedures in the United States suggest that 80% of patients experienced acute pain after surgery and state that postoperative pain continues to be undermanaged [18]. In addition, pain was shown to delay recovery in 24% of patients undergoing ambulatory surgery [19].

Reports on pain management approaches and outcomes in cancer palliative care patients in Lebanon and the Middle East have been published [20–26]. However, only few observational studies addressed the management of acute noncancer pain in Lebanese hospitals with a focus on physician implementation of pain management guidelines and practices [27, 28]. These studies reported the lack of well-structured systems for pain management in Lebanese hospitals and underlined the need for further research in the region [27, 28].

Accordingly, the primary objectives of this study were to assess (1) patients' description/rating of their acute pain intensity before and after surgical procedures; (2) patients' attitude and perception towards their pain management during hospitalization; and (3) their overall satisfaction with pain treatment. Secondary objectives were (1) to identify predictive factors that affect patients' satisfaction with pain management and (2) to evaluate the agreement between patients' overall satisfaction with their pain management and that of their healthcare providers.

## 2. Methods

### 2.1. Study Design and Setting

A prospective, cross-sectional study was conducted between October 2014 and March 2015 in three academic medical centers in Beirut, Lebanon. The study targeted two different in-patient adult populations who are most likely to experience acute noncancer pain: orthopedic surgery and obstetric postdelivery patients.

### 2.2. Tool for Data Collection

Two sets of questionnaires, one for the orthopedic surgery and another for the obstetric postdelivery patients, were developed by the investigators in English and then translated to Arabic by a licensed translator for use with patients. Questionnaires were developed in congruence with the American Pain Society Patient Outcome Questionnaire (APS-POQ) and modified to align with the study objectives [29]. The questionnaires were first pilot-tested, before administration, to ensure validity and clarity of included questions.

A brief cover letter was provided to participants, detailing the purpose of the questionnaire and reassuring confidentiality. Participating patients were asked to voluntarily and anonymously fill out the questionnaires that included the following sections: (1) demographics, (2) pain characteristics including intensity, (3) attitude towards pain management including patient expectations and preferences, and (4) patient satisfaction with pain management.

Demographic information included gender, age, marital status, educational and occupational status, type of healthcare coverage, and other demographics as applicable to the study population. Pain intensity was measured with the items “least” and “most” severe based on numerical rating scales (NRS) with answer options ranging from 0 to 10, where 0 reflects no pain and 10 worst pain possible. Pain severity was further categorized, as described by the patient, into the following categories: no pain (NRS score of 0), mild (NRS score of 1–3), moderate (NRS score of 4–6), severe but tolerable (NRS score of 7–9), and excruciating (NRS score of 10). Pain intensity was assessed right before and right after the procedure for both populations studied. For the purpose of this study, “right before” was defined as the day of the procedure, while “right after” was defined as day 1 after procedure. Similar to the APS-POQ, the questionnaire targeting the orthopedic surgery population assessed pain interference with activities and sleep, with answer options from 0 to 10, where a higher number indicates more interference [29]. The activity items addressed activities in bed (turning, repositioning) and activities out of bed (walking, sitting, and standing). The sleep items addressed difficulty falling asleep and difficulty staying asleep. Interference was further categorized, as reported by the patient, into the following categories: no interference (score of 0), mild interference (score of 1–3), moderate interference (score of 4–6), severe interference (score of 7–9), and debilitating interference (score of 10).

Attitude towards pain management section included questions related to (1) patients' barriers to receiving pain medications (fear of side effects, addiction/tolerance, and cost); (2) pain assessment by a healthcare provider prior to delivery of intervention; (3) patient education by a healthcare provider regarding pain treatment; and (4) timely delivery of intervention. Patient satisfaction was measured using a 4-point Likert scale including strongly dissatisfied, dissatisfied, satisfied, and strongly satisfied.

### 2.3. Data Collection

Third-professional-year pharmacy students from the Lebanese American University School of Pharmacy were properly trained by their clinical preceptor to approach patients asking for their willingness to fill out the questionnaire and to assist them in collecting the required data. Patients were eligible for inclusion if they were alert and have been hospitalized for at least 24 hours on the orthopedic surgery and obstetric postdelivery patient care services. Data were collected on day 1 after delivery or surgery. Upon completion of the questionnaire, students place the collected data forms in a sealed envelope that was then submitted to the primary investigator at the end of each week. The investigators did not have any control over the pain management delivery to the patients.

Data collection for the outcomes involving the healthcare providers (HCPs: physicians or nurses) was completed through the input of the healthcare providers who were in direct charge of patient care. This included a personal communication with the HCPs in charge to assess (1) their rating of patients' pain after the procedure, (2) their documentation of the patients' pain intensity in the medical chart, and (3) their overall satisfaction with the result of the pain management they provided.

No participants' identifiable information (name or contact information) was collected and all obtained information was processed confidentially. The study was approved by the Lebanese American University Institutional Review Board and the applicable executives of the three involved hospitals. The study was performed in accordance with the ethical standards as laid down in the 1964 Declaration of Helsinki and its later amendments.

### 2.4. Statistical Analysis

Completed questionnaires were entered in an excel form throughout the study duration using a coding system, and then data were analyzed using the SPSS version 21 software. Data from each unit were entered separately on different excel sheet to maintain accuracy of records of each unit thus avoiding mixing of data entry and analysis. Demographic data, information about pain intensity, and attitudes towards pain management were summarized using descriptive statistics. The association between categorical variables was evaluated using Pearson *χ*^2^ test or Fisher's exact test where the expected cell count was less than 5. *p* values below 0.05 were considered to be statistically significant.

## 3. Results

### 3.1. Primary Endpoints

#### 3.1.1. Participants' Characteristics

A total of 119 women on the maternity services and 177 patients on the orthopedic services were surveyed on day 1 after surgery. The majority of surveyed patients were married (100% and 76.3%) and completed high-school (33.6% and 31.6%) or university education (48.7% and 33.3%); were employed (64.7% and 49.7%); and were covered by private insurances (30.3% and 31.1%) or national social security fund (37.8% and 42.9%) in the maternity and orthopedic patient populations, respectively. Patients' sociodemographic characteristics are listed in Table 1.

#### 3.1.2. Pain Intensity

The majority of obstetric patients described their pain as mild to moderate at its least intensity (43.2% and 36.4%, resp.). When at its highest, the pain was mostly reported as moderate to severe (33.6% and 48.7%, resp.). Pain experienced right before delivery was generally severe (31%) or excruciating (37%) in this patient population. Right after delivering the baby, patients were still experiencing moderate (42%), severe (30.3%), and excruciating pain (13.4%) (Table 2).

Orthopedic patients varied in their description of pain at its least severity and reported pain of different intensities: mild (22.0%), moderate (36.7%), severe (21.55%), and excruciating (19.8%). When at its highest, the pain intensity was again broadly reported as moderate (18.1%), severe (36.7%), and excruciating (18.1%). Right before the surgical procedure, pain intensity in orthopedic patients was variable as follows: mild (20.9%), moderate (25.4%), severe (26%), and excruciating (27.1%). After the surgical procedure, patients still experienced variable pain severity with more of them reporting moderate (28.9%) and severe pain intensity (29.9%). After surgery, pain was moderately interfering with activities in bed such as turning and repositioning (44.6%) and activities out of bed such as walking or sitting in a chair or standing (46.9%) (Table 2).

Healthcare providers (HCPs) were asked to rate the patients' pain intensity right after the procedures. For both studied populations, HCPs mostly reported the pain as moderate in intensity: 19.3% for obstetric patients and 27.7% for orthopedic patients. Moreover, it is worth noting that pain scores were documented in obstetric and orthopedic patients' charts in 5% and 6.8% of the cases only, respectively (Table 2).

#### 3.1.3. Patients' Attitude/Perception towards Pain Management

Sixty percent of obstetric patients and 74.6% of orthopedic patients mentioned that they would like to be treated when in pain, while some of them preferred not to be treated (22.7% of obstetric patients versus 10.7% of orthopedic patients). Barriers to pain management identified by patients included fear of adverse reactions as the main barrier listed by both obstetric and orthopedic patients (52.1% and 49.2% resp.), followed by the fear of addiction potential (11.8% and 18.1%), tolerance (16% and 13%), and additional cost (12.4% and 13.6%) (Table 3).

Several unfavorable management practices related to pain assessment and management were reported in the obstetric and orthopedic services (Table 3). These included the following findings: (1) pain not being assessed prior to pain medication administration (19.3% and 30.5%, resp.); (2) patients not being asked about their previous use of medications for pain management (23.5% and 19.2%, resp.); (3) patients not being provided with sufficient education regarding the importance of pain reporting and management (21% and 14.7%, resp.); (4) patients not being informed when they were given medications for pain management (8.4% and 19.2%, resp.); (5) patients having to wait for more than 30 minutes before getting the pain medication when requested (14.6% and 11.3%, resp.); and (6) patients not being provided with appropriate atmosphere of quiet and peace to sleep at night (5% and 12.4%, resp.).

#### 3.1.4. Patients' Satisfaction with Pain Management

When asked about satisfaction with the overall pain management, the majority of patients in obstetric and orthopedic services reported to be satisfied (67.2% and 59.3%, resp.) to strongly satisfied (26.9% and 29.9%, resp.). Generally, 5.8% of the obstetric patients and 10.7% of the orthopedic patients were dissatisfied (dissatisfied and strongly dissatisfied) with the overall pain management they received (Table 3).

### 3.2. Secondary Endpoints

#### 3.2.1. Predictive Factors Associated with Patients' Satisfaction with Pain Management

Results detailing the sociodemographic factors and their association with patients' satisfaction with pain management for the combined populations of obstetric and orthopedic patients are presented in Table 4. These results revealed that gender, age, and educational and occupational status were not statistically significant patient-related predictive factors for satisfaction with pain management. However, being single (14.3% versus 7.9%; *p* = 0.041), not having Ministry of Health coverage (9.2% versus 4.2%; *p* = 0.025), and not receiving first class services were associated with higher patients dissatisfaction (Table 4).

Similarly, pain management related factors were studied for their association with patients' satisfaction for the combined populations of obstetric and orthopedic patients and presented in Table 5. Patients who feared additional costs ensuing from treatment had a statistically significant higher dissatisfaction with pain management compared to those with no such fear (*p* = 0.030). Patients who were not asked about their previously used pain medication (17.7% versus 6.4%, *p* = 0.008), those who were not questioned about their pain severity prior to medication administration (17.3% versus 5.5%, *p* = 0.006), and those who did not receive timely medication administration (<30 minutes) (7.7% versus lower percentages, *p* < 0.001) were dissatisfied with the delivered care. In addition, failing to educate the patient about the importance of pain management and the need to report uncontrolled pain was identified as a predictive factor (Table 5).

#### 3.2.2. Agreement between Patients' Overall Satisfaction with Pain Management and Healthcare Providers' Satisfaction

Data from HCPs was collected for 44 patients out of 119 in the obstetric services and for 84 out of 177 patients in the orthopedics services. In both patient populations, HCPs showed an overall satisfaction with the delivered pain management. Healthcare providers' satisfaction was not significantly correlated with those of the studied patient populations (*p* > 0.05 for both populations) (Table 6).

## 4. Discussion

This study addressed patients' perception of pain management in two patient populations experiencing postoperative acute noncancer pain. Our results showed that pain was prevalent and consistently experienced by obstetric and orthopedic patients before and after surgical procedures in varying intensities. In fact, at least 50% of patients in each of the studied populations experienced severe to excruciating pain right before the procedure. Similarly, at least 40% of patients in each of the surveyed populations reported being in severe to excruciating pain right after the procedure. Such findings suggest that the problem of postoperative pain remains substantial despite the availability of treatment options and published guidelines for pain management. This is in line with published studies in obstetric patients showing that acute postdelivery pain was commonly reported [30, 31]. In their study on the effect of postoperative pain on breastfeeding and infant care, Karlström et al. found that 78% of surveyed women experienced moderate levels of pain during the first 24 hours after delivery [30]. In orthopedic patients, the results of studies showed that 40 to 70% of surgical patients experience moderate and severe postoperative pain [32, 33].

When asked about their pain intensity, our studied populations demonstrated a widespread subjectivity in reporting pain intensity that encompassed all pain intensity levels during the different assessment time points. This is in congruence with the definition of pain by the International Association for the Study of Pain whereby pain is referred to as an emotional experience [34]. This emotional experience can be highly subjective and vary substantially among individuals [35].

Pain intensity is the most relevant clinical dimension of the patients' pain experience [36, 37]. Validated and reliable methods for pain intensity measurement include visual analogue scales (VAS), numerical rating scales (NRS), and verbal rating scales (VRS) [38]. These subjective (self-report) measurement methods of pain have been proven to be highly intercorrelated, with none of them consistently shown to be superior to the others [39, 40]. In the current study, the pain intensity data was collected based on the NRS and reported based on the VRS. The time frame for pain intensity assessment was set at day 1 after procedure and collected at its least, its highest, right before procedure, and right after it. Pain ratings in the past 24 hours have been shown to be valid and utilized in similar patient populations [30, 41].

An intervention-necessitating finding in our current study is that documentation of pain intensity was not completed for more than 90% of surveyed patients according to their HCPs. Although 80% of obstetric patients and 69.5% of orthopedic patients confirmed that their pain was assessed prior to pain medication administration, documentation of that pain assessment was only completed in 5% of obstetric patients and 6.8% of orthopedic patients. Similarly, multiple studies on pain management have shown that documentation of pain assessment was not consistently done for the majority of patients [42–45]. Despite guidelines underpinning pain assessment as the cornerstone of pain management and its documentation as an essential tool to make pain more discernible, pain intensity documentation remains suboptimal [45, 46]. This calls for healthcare institutions to implement policies and procedures mandating continuous pain assessment and documentation.

Barriers to effective pain management reported in this study and based on the surveyed patients' perspective included fear of adverse effects followed by addiction, tolerance, and fear of cost. This lack of patients' knowledge regarding pain issues was also identified by the First National Pain Medicine Summit as one of the top three barriers to receiving adequate patient care along with lack of competent pain providers and lack of knowledge by peers and/or patients regarding the field of pain medicine. The Summit further details patients' barriers to include (1) belief that pain is inevitable; (2) reluctance in reporting pain and in taking prescribed medications; (3) concern about addiction and the adverse effects of medications; (4) inaccessibility of pain management professionals; and (5) cost of pain medications and lack of insurance coverage for pain management [47].

This study provided optimistic data suggesting that communication between patients in pain and HCPs was being implemented. Patients were engaged in the care process as 76.5% and 80.8% of obstetric and orthopedic patients, respectively, were asked about previously used medications for pain management. In addition, 63% and 67.2% of obstetric and orthopedic patients, respectively, were asked to report when in pain. Moreover, 80.7% and 74% of obstetric and orthopedic patients were informed when pain medications were administered. Accordingly, such favorable practices involving communication and patient engagement in the care process could explain our positive findings of patient satisfaction with the delivered care despite the substantial pain that was still being experienced. In fact, 94.1% of obstetric patients and 89.2% of orthopedic patients were satisfied to strongly satisfied with the pain management they received.

Similarly, Bourdillon et al. assessed patient perception of pain management in an effort to determine the factors related to patient satisfaction [48]. In their study, Bourdillon et al. reported that 81% of surveyed patients were satisfied with their pain management and identified patient satisfaction to be higher when doctors and nurses were heavily involved in pain management through pain intensity assessment, immediate provision of treatment, and encouragement to ask for an analgesic when pain is persistent [48]. Our findings are also supported by Zoëga et al. reporting that patient's participation in decision making regarding their pain management leads to better pain relief [7]. In fact, patients' engagement in their own care has been reported to improve satisfaction as demonstrated in patient controlled analgesia literature and more recently in McTier et al. [49–51].

A secondary study objective identifying predictive factors for patient satisfaction with pain management further supports the importance of patient engagement in the care process to ensure satisfaction. The practices in pain management were found to be statistically correlated with patient dissatisfaction in this study mostly centered on patients' engagement in their care. Dissatisfied patients were those who did not have their pain assessed prior to pain medication administration, those who were not asked about previous medications used for pain management, and those who were not informed to report it when in pain. In addition to involving patients in their own care, our study reported that the timely delivery of pain medications to the patients was another factor affecting patient satisfaction. These results are in congruence with the previously referenced literature on patient engagement and satisfaction with care which can be explained by the notion that both ideas stem from trust and communication between HCPs and patients [35].

Interestingly, an incongruity was noted among the HCPs' and the survey populations' satisfaction. In fact, HCPs' satisfaction was independent of patients' satisfaction levels. Such a disagreement in satisfaction with pain management was reported in the literature and related to various reasons. The latter include subjectivity of pain experience and the difficulty of accurately quantifying it; differing interpretations of patient pain intensity and treatment response between patients and HCPs; and a general perception that patients exaggerate their pain experience [8, 52, 53].

To our knowledge, no studies in Lebanon report on the pain intensity and attitude towards pain management in the postoperative setting from patients' perspective. This study addressed a current and essential clinical problem that remains suboptimally managed. The method is strongly elaborated and includes two large patient populations with different conditions, which strengthens our findings. A limitation in our study is that interviewed participants could have recall bias. Another limitation, in our secondary endpoints, was the missing information from the patients' charts and the relatively low number of healthcare providers that we were able to interview. Yet, this study was able to identify the urgent need to have HCPs document pain intensity scores in patients' charts and the importance of engaging patients to ensure their satisfaction with pain management. Our findings will be shared with the concerned institutions to better understand their patient population needs, contemplate the implementation of acute pain management services, and improve on pain assessment, management, and patient education.

## 5. Conclusion

Patients presenting with acute pain expect and deserve utmost treatment. High quality pain management should include appropriate assessment; collaborative care planning comprising patient involvement; and efficacious treatment resulting in overall patient satisfaction. Pre- and postoperative pain remain a prevalent and severe problem that requires a consensus and joint efforts for improvement. Identified patient barriers that hamper pain management must be overcome. Active patient participation in their care might be an effective way to improve pain management.

## Figures and Tables

**Table 1:** Sociodemographic characteristics.

Characteristic	Obstetrics (*N* = 119) *n* (%)	Orthopedics (*N* = 177) *n* (%)
Gender		
Male	0	85 (48.0%)
Female	119 (100%)	92 (52.0%)

Age category		
Less than 18 years	4 (3.5%)	3 (1.9%)
18 to 30 years	57 (50.0%)	21 (13.0%)
30 to 40 years	51 (44.7%)	31 (19.1%)
40 to 50 years	2 (1.8%)	31 (19.1%)
51 years and more	0	76 (46.9%)

Marital status		
Single	0	42 (23.7%)
Married	119 (100%)	135 (76.3%)

Educational status		
Less than high school education	5 (4.2%)	42 (23.7%)
High-school graduate	40 (33.6%)	56 (31.6%)
Some university education	16 (13.4%)	20 (11.3%)
University graduate	58 (48.7%)	59 (33.3%)

Occupational status		
Unemployed	27 (24.4%)	66 (37.3%)
Employed	77 (64.7%)	88 (49.7%)
Self-employed	13 (10.9%)	12 (6.8%)
Financially dependent (student)	—	11 (6.2%)

Health insurance coverage		
Self-payer	5 (4.2%)	12 (6.8%)
Private insurance coverage	36 (30.3%)	55 (31.1%)
National social security fund (NSSF) coverage	45 (37.8%)	76 (42.9%)
Ministry of Public Health (MOH) coverage	6 (5.0%)	19 (10.7%)

Service class		
First class	38 (31.9%)	41 (23.2%)
Second class	35 (29.4%)	29 (16.4%)

**Table 2:** Pain intensity rating.

Pain description according to the patient	Obstetrics (*N* = 119) *n* (%)	Orthopedics (*N* = 177) *n* (%)
How would you describe your pain when at *its least* severity?		
No pain	12 (10.2%)	0
Mild pain	51 (43.2%)	39 (22.0%)
Moderate pain	43 (36.4%)	65 (36.7%)
Severe pain	12 (10.2%)	38 (21.55%)
Excruciating pain	0	35 (19.8%)

How would you describe your pain when at *its highest* severity?		
No pain	0	0
Mild pain	5 (4.2%)	4 (2.3%)
Moderate pain	40 (33.6%)	32 (18.1%)
Severe pain	58 (48.7%)	65 (36.7%)
Excruciating pain	16 (5.4%)	32 (18.1%)
Missing information	—	44 (24.9%)

How would you describe your pain *right before* the surgical procedure?		
No pain	0	0
Mild pain	19 (16.0%)	37 (20.9%)
Moderate pain	19 (16.0%)	45 (25.4%)
Severe pain	37 (31.1%)	56 (26.0%)
Excruciating pain	44 (37%)	48 (27.1%)

How would you describe your pain *right after* the surgical procedure?		
No pain	0	0
Mild pain	17 (14.3%)	25 (14.1%)
Moderate pain	50 (42.0%)	51 (28.9%)
Severe pain	36 (30.3%)	53 (29.9%)
Excruciating pain	16 (13.4%)	23 (13.0%)

Pain description according to the healthcare providers (HCPs)	Obstetric HCPs *n* (%)	Orthopedic HCPs *n* (%)

How would you rate the patient's pain after the surgical procedure?		
No pain	—	5 (2.8%)
Mild pain	9 (7.6%)	12 (6.8%)
Moderate pain	23 (19.3%)	49 (27.7%)
Severe pain	12 (10.1%)	13 (7.3%)
Excruciating pain	—	5 (2.8%)
Missing information	75 (63%)	93 (52.5%)

Did you document the patient's pain intensity in the medical chart?		
No	113 (95%)	165 (93.2%)
Yes	6 (5.0%)	12 (6.8%)

**Table 3:** Patients' attitude towards pain management.

Attitude	Obstetrics (*N* = 119) *n* (%)	Orthopedics (*N* = 177) *n* (%)
Identified barriers to administration of medications for pain management:		
Fear of adverse effects	62 (52.1%)	90 (49.2%)
Fear of addiction potential	14 (11.8%)	32 (18.1%)
Fear of additional cost	16 (12.4%)	24 (13.6%)
Fear of tolerance	19 (16.0%)	23 (13.0%)

Generally when you are in pain, would you like to be treated?		
No	27 (22.7%)	19 (10.7%)
Yes	71 (59.7%)	132 (74.6%)
Depending on pain severity	21 (17.6%)	26 (14.7%)

Were you asked about previously used medications for pain management?		
No	28 (23.5%)	34 (19.2%)
Yes	91 (76.5%)	143 (80.8%)

Was your pain assessed prior to pain medication administration?		
No	23 (19.3%)	54 (30.5%)
Yes	96 (80.7%)	123 (69.5%)

Did a healthcare provider inform you that pain management is important and asked you to report when in pain?		
Yes	75 (63.0%)	119 (67.2%)
Yes, but not sufficient	25 (21.0%)	26 (14.7%)
No, though I wanted to know	14 (11.8%)	22 (12.4%)
No and I do not want to know	5 (4.2%)	10 (5.6%)

Were you informed every time pain medications were administered?		
No	10 (8.4%)	34 (19.2%)
Yes	96 (80.7%)	131 (74.0%)
Inconsistently	13 (10.9%)	12 (6.8%)

What was the longest time you had to wait to get a pain medication after asking for it?		
<10 min	51 (42.9%)	93 (52.5%)
10–30 min	36 (30.3%)	41 (23.2%)
30–60 min	16 (13.4%)	13 (7.3%)
>60 min	1 (0.8%)	7 (4.0%)
Asked but never received pain medication	0	2 (1.1%)
Never asked for pain medication	15 (12.6%)	21 (11.9%)

Were you provided with an appropriate atmosphere of peace and quiet to sleep at night?		
No	6 (5.0%)	22 (12.4%)
Yes	113 (95.0%)	155 (87.6%)

How would you rate your overall satisfaction with the pain management you received?		
Strongly dissatisfied	1 (0.8%)	3 (1.7%)
Dissatisfied	6 (5.0%)	16 (9.0%)
Satisfied	80 (67.2%)	105 (59.3%)
Strongly satisfied	32 (26.9%)	53 (29.9%)

**Table 4:** Sociodemographic predictive factors associated with patient's satisfaction with pain management (combined obstetric and orthopedic patients).

Characteristic	Strongly dissatisfied and dissatisfied *n* = 26	Satisfied *n* = 185	Strongly satisfied *n* = 85	*p* value	Total *N* = 296
Gender					
Male	8 (9.4%)	52 (61.2%)	25 (29.4%)	0.948	85
Female	18 (8.5%)	133 (63.0%)	60 (28.4%)		211

Age category					
Less than 18 years	1 (14.3%)	5 (71.4%)	1 (14.3%)		7
18 to 30 years	5 (6.4%)	48 (61.5%)	25 (32.1%)	0.408	78
30 to 40 years	6 (7.3%)	53 (64.6%)	23 (28.0%)		82
40 to 50 years	7 (21.2%)	19 (57.6%)	7 (21.2%)		33
51 years and more	7 (9.2%)	50 (65.8%)	19 (25.0%)		76

Marital status					
Single	6 (14.3%)	19 (45.2%)	17 (40.5%)	0.041	42
Married	20 (7.9%)	166 (65.4%)	68 (26.8%)		254

Educational status					
Less than high-school education	13 (12.6%)	60 (58.25%)	40 (29.1%)	0.348	103
High-school graduate	8 (8.1%)	67 (67.7%)	24 (24.2%)		99
Some university education	4 (11.1%)	22 (61.1%)	10 (27.8%)		36
University graduate	1 (1.7%)	36 (62.1%)	21 (36.2%)		58

Occupational status					
Unemployed	14 (14.7%)	47 (49.5%)	34 (35.8%)		95
Employed	10 (6.1%)	112 (67.9%)	43 (26.1%)	0.053	165
Self-employed	1 (4.0%)	18 (72.0%)	6 (24.0%)		25
Financially dependent	1 (9.1%)	8 (72.7%)	2 (18.2%)		11

Ministry of Health Coverage					
No	25 (9.2%)	174 (64.2%)	72 (26.6%)	0.025	271
Yes	1 (4.0%)	11 (44.0%)	13 (52.0%)		25

First-class service					
No	23 (10.6%)	125 (57.6%)	69 (31.8%)	0.012	217
Yes	3 (3.8%)	60 (75.9%)	16 (20.3%)		79

**Table 5:** Pain management predictive factors associated with patient's satisfaction (combined obstetric and orthopedic patients).

Characteristic	Strongly dissatisfied and dissatisfied *n* = 26	Satisfied *n* = 185	Strongly satisfied *n* = 85	*p* value	Total *N* = 296
Generally when you are in pain, would you like to be treated?					
No	3 (6.5%)	25 (54.3%)	18 (39.1%)	0.523	46
Yes	19 (9.4%)	131 (64.5%)	53 (26.1%)		203
Depending on pain severity	4 (8.5%)	29 (61.7%)	14 (29.8%)		47

Fear of adverse effects					
No	18 (12.5%)	84 (58.3%)	42 (29.2%)	0.074	144
Yes	8 (5.3%)	101 (66.4%)	43 (28.3%)		152

Fear of addiction					
No	21 (8.4%)	151 (60.4%)	78 (31.2%)	0.088	250
Yes	5 (10.9%)	34 (73.9%)	7 (15.2%)		46

Fear of additional cost					
No	20 (7.8%)	156 (60.9%)	80 (31.3%)	0.030	256
Yes	6 (15.0%)	29 (72.5%)	5 (12.5%)		40

Fear of tolerance					
No	22 (8.7%)	160 (63.0%)	72 (28.3%)	0.912	254
Yes	4 (9.5%)	25 (59.5%)	13 (31.0%)		42

Were you asked about previously used medications for pain management?					
No	11 (17.7%)	39 (62.9%)	12 (19.4%)	0.008	62
Yes	15 (6.4%)	146 (62.4%)	73 (31.2%)		234

Was your pain assessed prior to pain medication administration?					
No	13 (17.3%)	44 (58.7%)	18 (24.0%)	0.006	75
Yes	12 (5.5%)	141 (64.4%)	66 (30.1%)		219

Did a healthcare provider inform you that pain management is important and asked you to report when in pain?					
Yes	11 (5.7%)	115 (59.3%)	68 (35.1%)	<0.001	194
Yes but not sufficient	2 (3.9%)	37 (72.5%)	12 (23.5%)		51
No but I wanted to know	9 (25.0%)	24 (66.7%)	3 (8.3%)		36
No and I do not want to know	4 (26.7%)	9 (60.0%)	2 (13.3%)		15

What was the longest time you had to wait to get a pain medication after asking for it?					
<10 minutes	0	2 (100%)	0		2
10–30 minutes	11 (7.7%)	79 (55.6%)	52 (36.6%)	<0.001	142
30–60 minutes	4 (5.2%)	66 (85.7%)	7 (9.1%)		77
>60 minutes	3 (10.3%)	23 (79.3%)	3 (10.3%)		29
Asked but never received	5 (62.5%)	1 (12.5%)	2 (25.0%)		8
Never asked for pain medication	1 (2.8%)	14 (38.9%)	21 (58.3%)		36

Were you provided with an appropriate atmosphere of peace and quiet to sleep at night?					
No	8 (28.6%)	13 (46.4%)	7 (25.0%)	<0.001	28
Yes	18 (6.7%)	172 (64.2%)	78 (29.1%)		268

**Table 6:** Agreement between the Healthcare Providers' Satisfaction and Patients' Declared Satisfaction.

Obstetrics	Healthcare providers' satisfaction (*N* = 44)			
	Strongly dissatisfied and dissatisfied *n* = 2	Satisfied *n* = 39	Strongly satisfied *n* = 3	*p* value
Patients' satisfaction				
Strongly dissatisfied and dissatisfied (*n* = 2)	0	2 (100%)	0	1.000
Satisfied (*n* = 30)	2 (6.7%)	26 (86.7%)	2 (6.7%)	
Strongly satisfied (*n* = 12)	0	11 (91.7%)	1 (8.3%)	

Orthopedics	Healthcare providers' satisfaction (*N* = 84)			
	Strongly dissatisfied and dissatisfied *n* = 5	Satisfied *n* = 69	Strongly satisfied *n* = 10	*p* value

Patients' satisfaction				
Strongly dissatisfied (*n* = 2)	0	2 (100%)	0	0.594
Dissatisfied (*n* = 9)	1 (11.1%)	8 (88.9%)	0	
Satisfied (*n* = 55)	3 (5.5%)	46 (83.6%)	6 (10.9%)	
Strongly satisfied (*n* = 18)	1 (5.6%)	13 (72.2%)	4 (22.2%)	
